# Novel CTRP8‐RXFP1‐JAK3‐STAT3 axis promotes Cdc42‐dependent actin remodeling for enhanced filopodia formation and motility in human glioblastoma cells

**DOI:** 10.1002/1878-0261.12981

**Published:** 2021-06-18

**Authors:** Aleksandra Glogowska, Thatchawan Thanasupawat, Jason Beiko, Marshall Pitz, Sabine Hombach‐Klonisch, Thomas Klonisch

**Affiliations:** ^1^ Department of Human Anatomy and Cell Science Rady Faculty of Health Sciences College of Medicine University of Manitoba Winnipeg Canada; ^2^ Department of Surgery Rady Faculty of Health Sciences College of Medicine University of Manitoba Winnipeg Canada; ^3^ Research Institute in Oncology and Hematology (RIOH) CancerCare Manitoba Rady Faculty of Health Sciences College of Medicine University of Manitoba Winnipeg Canada; ^4^ Department of Medical Microbiology & Infectious Diseases Rady Faculty of Health Sciences College of Medicine University of Manitoba Winnipeg Canada; ^5^ Department of Pathology Rady Faculty of Health Sciences College of Medicine University of Manitoba Winnipeg Canada

**Keywords:** actin remodeling, Cdc42, CTRP8, glioblastoma, relaxin, RXFP1

## Abstract

C1q tumor necrosis factor‐related peptide 8 (CTRP8) is the least studied member of the C1Q‐TNF‐related peptide family. We identified CTRP8 as a ligand of the G protein‐coupled receptor relaxin family peptide receptor 1 (RXFP1) in glioblastoma multiforme (GBM). The CTRP8‐RXFP1 ligand–receptor system protects human GBM cells against the DNA‐alkylating damage‐inducing temozolomide (TMZ), the drug of choice for the treatment of patients with GBM. The DNA protective role of CTRP8 was dependent on a functional RXFP1‐STAT3 signaling cascade and targeted the monofunctional glycosylase *N*‐methylpurine DNA glycosylase (MPG) for more efficient base excision repair of TMZ‐induced DNA‐damaged sites. CTRP8 also improved the survival of GBM cells by upregulating anti‐apoptotic BCl‐2 and BCL‐XL. Here, we have identified Janus‐activated kinase 3 (JAK3) as a novel member of a novel CTRP8‐RXFP1‐JAK3‐STAT3 signaling cascade that caused an increase in cellular protein content and activity of the small Rho GTPase Cdc42. This is associated with significant F‐actin remodeling and increased GBM motility. Cdc42 was critically important for the upregulation of the actin nucleation complex N‐Wiskott–Aldrich syndrome protein/Arp3/4 and actin elongation factor profilin‐1. The activation of the RXFP1‐JAK3‐STAT3‐Cdc42 axis by both RXFP1 agonists, CTRP8 and relaxin‐2, caused extensive filopodia formation. This coincided with enhanced activity of ezrin, a key factor in tethering F‐actin to the plasma membrane, and inhibition of the actin filament severing activity of cofilin. The F‐actin remodeling and pro‐migratory activities promoted by the novel RXFP1‐JAK3‐STAT3‐Cdc42 axis were blocked by JAK3 inhibitor tofacitinib and STAT3 inhibitor STAT3 inhibitor VI. This provides a new rationale for the design of JAK3 and STAT3 inhibitors with better brain permeability for clinical treatment of the pervasive brain invasiveness of GBM.

## Graphical abstract

The C1Q‐TNF‐related peptide family member C1q tumor necrosis factor‐related peptide 8 (CTRP8) and its G protein‐coupled receptor relaxin family peptide receptor 1 (RXFP1) are emerging as a novel ligand–receptor system that can protect glioblastoma cells against DNA‐alkylating damage and apoptosis. We describe a novel CTRP8‐RXFP1‐Janus‐activated kinase 3‐STAT3 signaling cascade that enhances cellular protein content and activity of small Rho GTPase Cdc42, which directs F‐actin cytoskeletal remodeling, filopodia formation, and increased glioblastoma multiforme migration.

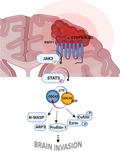

**Abbreviations**
CTRP8: C1q tumor necrosis factor‐related peptide 8
ERM: ezrin, radixin, and moesin
GBM: glioblastoma multiforme
JAK3: Janus‐activated kinase 3
RLN2: relaxin‐2
RXFP1: relaxin family peptide receptor 1
S3I‐201: STAT3 inhibitor VI
SIM: structured illumination microscopy
WASP: Wiskott–Aldrich syndrome protein

## 1 Introduction

Glioblastoma multiforme (GBM) is a high‐grade astrocytoma, constitutes > 50% of all malignant glioma in adults, and has the worst prognosis among all brain tumors with an average survival time of approx. 15 months after diagnosis [1, 2, 3]. Current treatment of GBM is palliative at best and consists of surgical resection followed by radiation and chemotherapy [4, 5, 6]. A hallmark of all malignant gliomas is the extensive brain tissue infiltration by GBM cells, which severely limits treatment success and results in frequent incurable and fatal GBM recurrences [7, 8].

The tissue invasion of GBM cells involves coordinated dynamic cytoskeletal changes in actin filaments and the production and secretion of proteolytic enzymes capable of digesting ECM matrices [9]. The activation of Rho small GTPase family members is a trademark for increased motility and tissue invasiveness of tumor cells [10, 11, 12]. Rho small GTPases are essential molecular regulators of dynamic actin filament remodeling [13, 14], and of the eight members of this family, RhoA, Rac1, and Cdc42 are the most studied with respect to their roles in cytoskeletal actin filament regulators of cell shape and movement [15, 16]. The regulation and function of Rho small GTPase family members depend on GTP‐to‐GDP hydrolysis, and the induced protein conformational changes are regulated by the coordinated actions of guanine nucleotide exchange factors (GEFs), GTPase‐activating proteins (GAPs), and guanine nucleotide dissociation inhibitors (Rho GDIs) [17, 18]. In their active form, small GTPases bind to phospholipids at the inner side of the cell membrane enabling them to stimulate downstream signaling pathways that mediate the diverse functions of Rho family members [17, 19, 20, 21]. All Rho small GTPases share common growth‐promoting and anti‐apoptotic functions and regulate gene expression through the activation of signaling molecules, such as serum response factor, NF‐κB, the stress‐activated protein kinases, and cyclin D1 [22, 23]. Rho family members are best known for their impact on the cell cytoskeleton and the ability to induce specific actin filamentous cellular phenotypes [24, 25, 26]. Each activated Rho member has unique functions that confer different cellular morphology and motility responses. RhoA signaling activates the serine/threonine kinase p160 ROCK to promote contractile actomyosin stress fiber formation [27] by inhibiting MLC phosphatase, thereby phosphorylating myosin light chain (MLC) [28, 29, 30]. Decreased RhoA activity was reported during glioma cell migration [31, 32]. Rac1 promotes the assembly of a peripheral actin meshwork, including lamellipodia formation [17, 33, 34]. Inhibition of ROCK leads to Rac 1 activation in glioma cells and promotes invasion, which coincided with increased membrane ruffling and a collapse of actin stress fibers [35]. The Rho small GTPase member Cdc42 causes the formation of filopodia or spikes. Bradykinin‐induced Cdc42 activation and filopodia formation regulate neural growth cone development and migration [36]. Cdc42 stimulates the formation of filopodia through its association with Wiskott–Aldrich syndrome protein (WASP) or N‐WASP [37]. Cdc42‐mediated filopodia formation is important for cancer cell migration, and dysregulated guanine nucleotide exchange factors (GEFs) Etc2 and Trio impact on Cdc42 and Rac1 activity, respectively. In a glial fibrillary acidic protein (GFAP)‐*tva* transgenic mouse model, astrocyte‐specific aberrant expression of guanine nucleotide exchange factor (GEF) Etc2 resulted in concordant dysregulation of Cdc42 activity affecting astrocyte cell proliferation and motility [38].

In human gliomas, Cdc42 is frequently overexpressed and Cdc42 expression correlates with higher glioma grade and poor prognosis for the overall survival of glioma patients [39]. Activated Cdc42 has been recognized as a critical mediator for an enhanced migratory and invasive phenotype of malignant gliomas [40, 41, 42].

The activation of the G protein‐coupled relaxin family peptide receptor 1 (RXFP1) promotes tumor growth, angiogenesis, migration, and tissue invasion in several human tumor types, including breast, thyroid, prostate, endometrium, and brain cancer [43, 44, 45, 46, 47, 48]. Targeted disruption of RXFP1 expression in subcutaneous PC3 cell xenografts significantly reduced cancer growth and invasiveness in mice. [49, 50] The classical RXFP1 agonist relaxin‐2 (RLN2) increases the invasiveness of breast cancer cell lines SK‐BR3, MCF7, and MDA‐MB‐231, [51, 52, 53, 54] thyroid cancer cell lines FTC133 and UTC8305, [55, 56] and prostate cancer cell lines LNCaP and PC3. [49, 50] This increased tumor cell motility coincides with altered cellular deposition of ECM components [46, 55, 57] and increased production/secretion of ECM‐degrading members of the matrix metalloproteinases MMP2, MMP3, and MMP9 [58] and cathepsin family, including cathepsin B in patient GBM cells [45, 55]. RXFP1 activation by the secreted adiponectin paralog and RXFP1 agonist C1q tumor necrosis factor‐related peptide 8 (CTRP8) resulted in elevated intracellular cAMP levels and activated PKCζ and PKCδ isoforms in human malignant glioma [45]. We showed that a CTRP8‐RXFP1‐STAT3 signaling axis protects GBM cells against alkylating DNA base damage of DNA‐alkylating agent temozolomide (TMZ), the standard chemotherapeutic drug for the treatment of GBM patients. This CTRP8‐RXFP1‐induced TMZ chemoresistance involved the upregulation of DNA‐3‐methyladenine glycosylase (MPG), which promotes DNA base excision repair [59].

Here, we have identified a new CTRP8/RLN2‐RXFP1‐Janus‐activated kinase 3 (JAK3)‐STAT3‐Cdc42 signaling axis as a critical determinant of GBM invasive phenotype. RXFP1 activation triggered the upregulation of Cdc42 protein and activation of the Cdc42‐WASP‐Arp2/3 cascade, which resulted in pronounced filopodia formation and motility of patient GBM cells.

## 2 Materials and methods

### 2.1 Primary human brain isolation and cell culture

Surgically removed GBM tissues were obtained from GBM patients treated at the local Health Science Centre, Winnipeg. The study methodologies conformed to the standards set by the Declaration of Helsinki. The study was approved by the University and Pathology ethics boards (ethics approval H2010:116), and patient consent was obtained prior to sample collection. Patient GBM cells were cultured in DME/F12 media plus 10% FBS and growth at 37 °C in a humidified 5% CO_2_ atmosphere incubator. The medium was changed to DME/F12 with 1% FBS 24 h prior to treatments. We use three different patient GBM cell models (GBM10, GBM34, and GBM146) for the experiments presented in this study. U87MG glioma cell line was cultured in DME/F12 media plus 10% FBS and growth at 37 °C in a humidified 5% CO_2_ atmosphere incubator.

### 2.2 Inhibitors and gene silencing

STAT3 inhibitor VI (S3I‐201) and JAK3 inhibitor (tofacitinib) were obtained from EMD Millipore (Billerica, MA, USA). Cells were pre‐incubated with either 30 µm of S3I‐201 or 10 µm of tofacitinib for 60 min prior to adding human recombinant CTRP8 and with RLN2 for selected experiments. ON‐TARGET plus SMART pool human RXFP1, Cdc42, and control siRNAs were purchased from Dharmacon (Dharmacon, Thermo Scientific, Waltham, MA, USA). The siLentFect lipid reagent (Bio‐Rad, Mississauga, ON, Canada) was used to transfect siRNAs into patient GBM cells at 5 × 10^4^ cells per well grown in six‐well plates. For the detection of RXFP1 and Cdc42 mRNA expression, total RNA was extracted and upon reverse transcription by RT‐PCR (Thermo Fisher, Walthman, CA, USA) and quantitative real‐time PCR (qPCR) with the following primers: RXFP1 forward: AAAAGAGATGATCCTTGCCAAACG, RXFP1 reverse: CCACCCAGATGAATGATGG AGC; GAPDH forward: CATCACCATCTTCCAGGAGCG, GAPDH reverse: TGACCTTGCCC ACAGCCTTG; and Cdc42 forward: TGCTGATCACTGTTAGAAATAACTCCTG, Cdc42 reverse: TCCTTTCTTGCTTGTTGGGACT. qPCR was performed with a QuantStudio® 3 system (Applied Biosystems, Ottawa, ON, Canada). The comparative C_T_ (ΔΔC_T_) method was used for data analysis using QuantStudio® Design & Analysis software. Samples were normalized to GAPDH expression. DMSO (Sigma‐Aldrich, Oakville, ON, Canada) was used to reconstitute S3I‐201 and tofacitinib inhibitors. Cells were treated with the same concentration of DMSO alone to account for solvent effects.

### 2.3 Phalloidin staining

Patient GBM cells were seeded on coverslips in six‐well tissue plates at 5 × 10^4^ cells in 10% FBS DME/F12 medium. Cells were exposed to medium with 1% FBS 24 h prior treated with 100 ng·mL^−1^ CTRP8 and RLN2 as a control. After 24 h, cells were fixed with 3.7% formaldehyde for 30 min followed by permeabilization using 0.1% Triton X‐100 dissolved in double‐distilled water prior to phalloidin–Alexa Fluor 594 staining (Thermo Fisher). DAPI (Sigma‐Aldrich) was used as a nuclear marker. Cells were imaged with a 63× and 40× objective and a Z2 microscope using ZEN imaging software (Zeiss, Jena, Germany).

Cells were imaged by super‐resolution structured illumination microscopy (SIM, Elyra, Zeiss) and processed with ZEN imaging software (Zeiss) to establish the number of filopodia in GBM cells. Experiment was repeated three times, and 50 cells were imaged and analyzed for CTRP8‐treated and nontreated cells, respectively. Cells were imaged at 40× and 63× magnification, and membrane extension was counted in a 1‐mm^2^ plasma membrane area.

### 2.4 Recombinant proteins

Recombinant human relaxin (RLN2) was generously provided by Corthera Inc. (San Mateo, CA, USA). Recombinant human CTRP8 was produced in *Escherichia coli* containing Flag‐tagged CTRP8 in pET28a as described previously [59].

### 2.5 Western blots

Proteins were separated on 12% or 10% SDS/PAGE and transferred to nitrocellulose or PVDF membranes. For immunodetection, nonspecific protein binding sites were blocked by incubation with 5% nonfat milk in TBS/T for 1 h at room temperature (RT). The following primary antibodies were all used at 1 : 1000 and incubated at 4 °C overnight from Cell Signaling Technologies (Danvers, MA, USA): pSTAT3^Tyr705^ (D3A7; #9145), pSTAT3^Ser727^ (D8C2Z; #94994), total STAT3 (D1A5; #8768), pJAK3^Tyr980/981^ (D44E3; #5031), total JAK3 (D1H3; #8827), Cdc42 antibody #4651, ARP3 antibody #4738, WAVE‐2 (D2C8; #3659), profilin‐1 (C56B8; #3246), N‐WASP (30D10; #4848), ezrin (#3142), phospho‐ezrin^T567^ (48G2; #3726), phospho‐cofilin^S3^ (77G2; #3313), cofilin (D3F9; #5175), phospho‐VASP^S157^ (#3111), VASP (9A2; #3132), fascin (D1A8; #9269), and 1 : 10 000 for β‐actin (Sigma‐Aldrich). Phosphorylated N‐WASP^Ser484/485^ protein was detected with the Antibody Sample Kit by ECM Bioscience (Versailles, KY, USA). Membranes were washed 3× for 5 min each in TBS/T (Tris base, NaCl, Tween‐20, all from Sigma‐Aldrich, CA, USA) at RT before incubating with horseradish peroxidase (HRP)‐conjugated secondary antibodies for 1 h at RT. Specific binding was visualized with clarity ECL substrate by ChemiDoc Imaging MP system (Bio‐Rad). Proteins with sufficiently different molecular weights were detected within the same membrane; this was the case for ARP3 (47 kDa)/Cdc42 (21 kDa) and profilin‐1 (15 kDa)/N‐WASP (65 kDa). Once phosphorylated proteins had been detected, membranes were stripped using 20% SDS with β‐mercaptoethanol in Tris buffer and reprobed for total protein detection. Beta‐actin was used as a loading control. Relative intensities of proteins detected by western blot were measured using the Bio‐Rad image lab software. Beta‐actin values were used to normalize protein levels. All densitometry graphs represent cumulative results from at least three independent experiments.

### 2.6 xCELLigence® real‐time cell analysis

We performed xCELLigence real‐time cell migration and viability assays (ACEA Biosciences, Inc., San Diego, CA, USA). Patient GBM cells were cultured on E‐plates (viability) and CIM plates (migration) treated with CTRP8, siRXFP1, siCdc42, si control, STAT3 inhibitor (S3I‐201), JAK3 inhibitor (tofacitinib), and DMSO solvent control. Changes in cellular impedance were represented as cell index (CI) and recorded every 15 min for 24 h upon treatment using the real‐time cell analysis (RTCA) software. Each migration assay was repeated three times in independent experiments.

### 2.7 Cdc42 activity assay

U87 cells were plated in 10‐cm diameter Petri dishes at 700 000 cells. After 24 h, the cells were washed once in 1× PBS and supplied with standard media containing 1% FBS. The next day, cells were incubated with 100 ng CTRP8 for 5, 10, 20, 30, and 60 min in media with 1% FBS. Incubation was stopped with ice‐cold 1XPBS. Cells were scraped and centrifuged at 800 r.p.m. for 5 min. Cell pellets were placed on ice, and lysis buffer was added. Lysis buffer was provided by the Cdc42 Activity Kit (New England BioLab, Whitby, ON, Canada). Experiments were done according to the manufacturer's protocol. Briefly, upon protein isolation, protein concentration was determinate by Pierce™ BCA Protein Assay Kit (Thermo Scientific) and 500 μg of total protein was used for each sample. Lysis buffer was mixed with 20 µg of GST‐PAK1‐PBD and added into the spin cup containing the glutathione resin. Reactions were incubated at 4 °C for 1 h. After washes, the mixture was centrifuged and samples were run for western blot analysis using a Cdc42 antibody.

### 2.8 Statistical analysis

All experiments were done at least in triplicate. Results are shown as mean ± standard deviation (SD). Data were analyzed with graphpad prism (San Diego, CA, USA) 6 statistical software using one‐way and two‐way ANOVA. *P*‐values less than 0.05 were considered significant. The level of significance was defined as *P < 0.05, **P < 0.01, ***P < 0.001, and ****P < 0.0001.

## 3 Results

### 3.1 A new CTRP8‐RXFP1‐JAK3‐STAT3‐Cdc42 signaling axis in human GBM cells

**Fig. 1:**
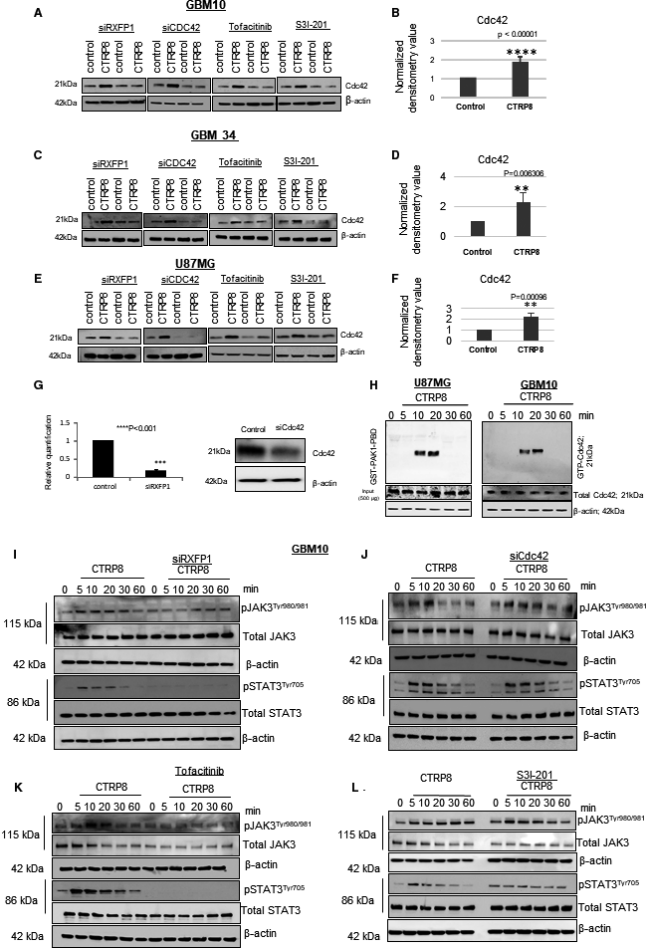
CTRP8 utilizes a new RXFP‐JAK3‐STAT3 axis for increased Cdc42 protein and activity in GBM cells. Western blot detection of Cdc42 in patient GBM10 (A), GBM34 (C), and U87MG (E) cells upon 24‐h stimulation with CTRP8 in the presence and absence of siRXFP1, siCdc42, JAK3 inhibitor tofacitinib, and STAT3 inhibitor S3I‐20. In all three GBM models, CTRP8 treatment increased the cellular Cdc42 protein content by approx. 40–50% compared with nontreated control, as quantified by densitometry of eight independent experiments for each model (*n* = 8), including GBM10 (B), GBM34 (D), and U87MG (F). Densitometry graphs represent mean with SD. Two‐tailed *t*‐test was used to determine the *P*‐value, and *P* < 0.05 was considered significant, and *P*‐values are indicated. qPCR analysis was used to show successful siRXCFP1‐mediated reduction of RXFP1 transcripts (G). Western blot detection of Cdc42 showed successful reduction in Cdc42 protein upon siCdc42 treatment (G). Detection of GTP‐bounded (active) Cdc42 in U87MG and GBM10 cells (H). GBM10 and U87MG cells treated with CTRP8 for 0–60 min were incubated with GST‐PAK1‐PDP, and PAK1‐interacting partners were pulled down with glutathione resins at a total protein input of 500 μg. Employing a specific Cdc42 antibody, we detected significant levels of PAK1‐interacting partner GTP‐Cdc42 at 10 and 20 min in GBM10 and U87MG by western blot (H). Western blot detection of pJAK3^Tyr980/981^, total JAK3, pSTAT3^Tyr705^, and total STAT3 in GBM10 upon CTRP8 treatment for 0, 5, 10, 20, 30, and 60 min (I–L). Phosphorylation of both JAK3 and STAT3 was diminished in GBM cells treated with CTRP8 and siRXFP1 KD (I), whereas phosphorylation of JAK3 and STAT3 was observed upon siCdc42 treatment (J). GBM cells co‐treated with CTRP8 and tofacitinib showed diminished pJAK3^Tyr980/981^ and pSTAT3^Tyr705^ phosphorylation (K), while co‐treatment with CTRP8 and S3I‐201 abolished STAT3 phosphorylation but failed to inhibit JAK3 phosphorylation (L). Our results identified a new CTRP8‐RXFP1‐JAK3‐STAT3‐Cdc42 axis. Beta‐actin was used as a loading control in all western blots (A, C, E, G, H–L).

**Fig. 2:**
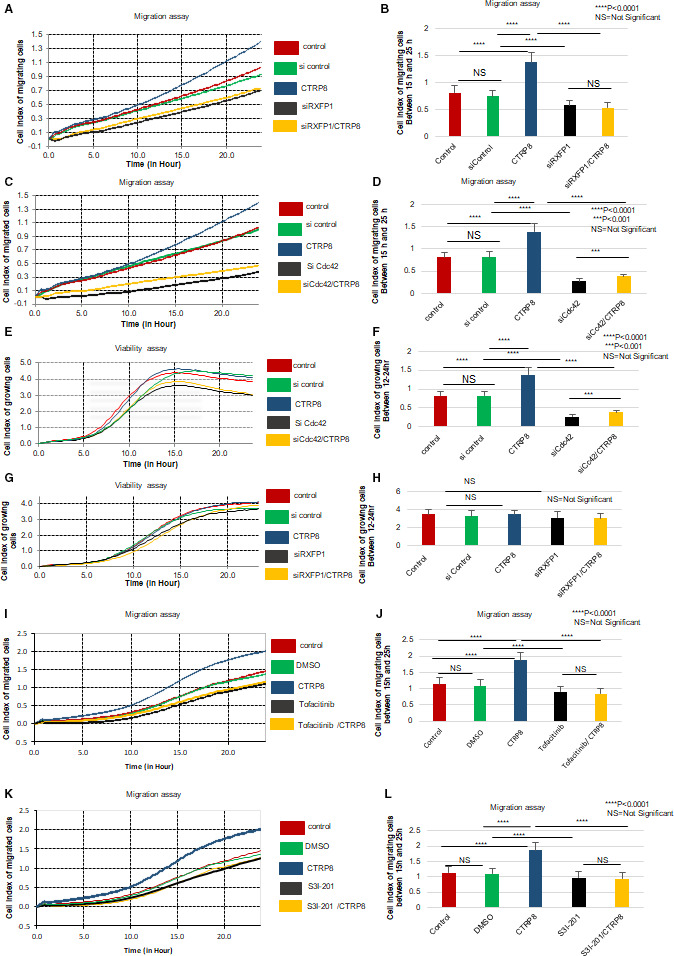
Activation of the RXFP1‐JAK3‐STAT3‐Cdc42 axis increases GBM motility. Representative real‐time migration curves of GBM10 motility measured by the RTCA‐DP system using CIM plates. Experiments were performed for up to 25 h, and measurements were recorded every 15 min. Cumulative measurement graphs (15–25 h) showed an average of 70% induction of GBM10 motility upon CTRP8 treatment (B, D, J, L). The enhanced migratory response of patient GBM cells to CTRP8 was abolished in the presence of siRXFP1 (A, B) and siCdc42 (C, D). The siCdc42 treatment selectively and significantly reduced GBM cell viability as measured by real‐time RTCA‐DP system using E‐plates. This effect was not observed with control siRNA. Treatment with siCdc42 was nontoxic and did not affect cell viability (E, F). Silencing of RXFP1 in GBM10 cells did not change the level of cell viability as measured by RTCA‐DP system. Treatment with control siRNA had no effect on GBM cell migration (A–D) or viability (G, H). Treatment with JAK1/3 inhibitor tofacitinib (10 µm; I, J) and STAT3 inhibitor S3I‐201 (30 µm; K, L) abolished the CTRP8‐mediated increase in GBM motility. Each experiment was performed as three independent duplicates for each treatment. ANOVA was used to determine significance by calculating four measurements for each experiment; results are displayed using graphpad prism. Data are represented as a mean values and SD with a *P*‐value of <0.001 (***) and < 0.00001 (****); NS, not significant.

Treatment of three different patient‐derived GBM cell models (GBM10, GBM34, GBM146) and the glioma cell line U87MG with human recombinant CTRP8 consistently resulted in a 40–50% upregulation of Cdc42 protein. This increase in Cdc42 protein was critically dependent on the presence of RXFP1 and abolished upon selective siRXFP1 knockdown (KD), while basal Cdc42 protein levels in patient GBM and U87MG cells remained unchanged by RXFP1 silencing alone or in combination with CTRP8 treatment (Fig. 1A–F). CTRP8 treatment was unable to rescue the targeted Cdc42 KD (Fig. 1A–G). The increase in total Cdc42 protein content by RXFP1 agonists CTRP8 (Fig. 1A–F) and RLN2 (Fig. S1G) included a temporary increase in active GTP‐Cdc42 at 10 and 20 min shown for treatment with CTRP8 in GBM10 and U87MG as determined by GTPase pull‐down assays (Fig. 1H). In the different GBM models studied, we confirmed our previous finding that RXFP1 agonists induced phosphorylated STAT3 at residue Y705 and showed that this STAT3^Tyr705^ phosphorylation was abolished upon RXFP1 KD, as demonstrated for CTRP8 (Fig. 1I–L; Fig. S1A,C) and RLN2 (Fig. S1H) [45, 59]. Next, we investigated the Janus‐activated kinase (JAK) signaling factors, which are known to facilitate signal transduction events by several cytokine receptors and G protein‐coupled receptors upstream of their phosphorylation target STAT3 [60, 61]. Of the three JAK members (JAK1–JAK3) present in the GBM models (data not shown), CTRP8 caused exclusive phosphorylation of JAK3 at residues Y890/891 (Fig. 1I–L; Fig. S1A). As for pSTAT3^Tyr705^, CTRP8‐mediated JAK3^Y890/891^ phosphorylation was dependent on RXFP1 (Fig. 1I; Fig. S1A). The specific KD of Cdc42 did not affect CTRP8‐mediated JAK3 and STAT3 phosphorylation (Fig. 1J; Fig. S1D). In the presence of CTRP8, both the JAK1/3 inhibitor tofacitinib (Fig. 1K; Fig. S1E) and STAT3 inhibitor S3I‐201 (Fig. 1L; Fig. S1B,F) were able to block the upregulation of pJAK3^Tyr890/891^ and pSTAT3^Tyr705^, respectively, while having no effect on cellular levels of total JAK3 and STAT3 proteins. S3I‐201 successfully inhibited CTRP8‐mediated pSTAT3^Tyr705^ phosphorylation but failed to block JAK3^Tyr890/891^ phosphorylation (Fig. 1L), indicating that JAK3 was upstream of STAT3 and excluding the possibility of a JAK3‐STAT3 signaling feedback loop [62, 63]. While tofacitinib and S3I‐201 did not affect basic levels of Cdc42 protein, both inhibitors blocked the upregulation of Cdc42 in response to CTRP8 treatment in human glioma cells (Fig. 1A,C,E). When treated with the same concentration of DMSO alone or scrambled control siRNA to account for solvent or siRNA effects, respectively, GBM cell responses were similar to untreated cells (Fig. S2A–E). For siRNA‐mediated Cdc42 and RXFP1 experiments, efficient KD was confirmed by PCR in the four patient GBM cell models used in this study (Fig. 2F,G). In summary, our results identified a new CTRP8‐RXFP1‐JAK3‐STAT3‐Cdc42 signaling axis, which resulted in the upregulation of active Cdc42 protein in human GBM cells.

### 3.2 The RXFP1‐Cdc42 axis promotes migration of patient GBM cells

In real‐time migration assays, GBM10/34/146 cells treated with CTRP8 responded with a significant increase in migration rate as compared to untreated cells (Fig. 2A–D,I,L; Fig. S3A–J). This CTRP8‐mediated increase in GBM motility was abolished by RXFP1 KD (Fig. 2A,B; Fig. S3A,B,E,F). A representative qPCR result of siRXFP1 KD is shown for GBM10 (Fig. 1G; for other GBM models, see Fig. S2F,G). Similarly, treatment of GBM10 with RLN2 also resulted in a significant increase in motility that coincided with strong pSTAT3^Tyr705^ and blocked by siRXFP1 KD (Fig. S3I,J). Next, we assessed the effect of increased Cdc42 protein production on GBM cell migration upon specific Cdc42 siRNA treatments. CTRP8‐mediated increase in GBM motility was abolished with the Cdc42 KD (Fig. 2C,D; Fig. S3C,D,G,H). A representative western blot of siCdc42‐mediated KD of Cdc42 protein levels is shown for GBM10 (Fig. 2G). Treatment of all patient GBM cells with siCdc42 caused efficient Cdc42 silencing (Fig. S1G). The effects on GBM migration observed upon treatment with CTRP8 or siCdc42 did not alter cell proliferation in our GBM models, as shown by real‐time viability assays for GBM10 (Fig. 2E,F) and WST metabolic assays (Fig. S3K). We used the JAK1/3 inhibitor tofacitinib and STAT3 inhibitor S3I‐201 to assess the involvement of the JAK3‐STAT3 signaling pathway in the CTRP8‐mediated enhanced motility of GBM using real‐time migration assays. Coinciding with the ability of tofacitinib to block JAK3 phosphorylation (Fig. 1K; Fig. S1E), this JAK3 inhibitor muted the ability of CTRP8 to enhance GBM motility (Fig. 2I,J). Likewise, STAT3 inhibitor S3I‐201 blocked CTRP8‐mediated increase in GBM cell motility (Fig. 2K,L). These results identified a new pro‐migratory function of the CTRP8‐RXFP1‐JAK3‐STAT3‐Cdc42 cascade in GBM.

### 3.3 RXFP1 activation induces F‐actin filament remodeling and promotes filopodia formation

**Fig. 3:**
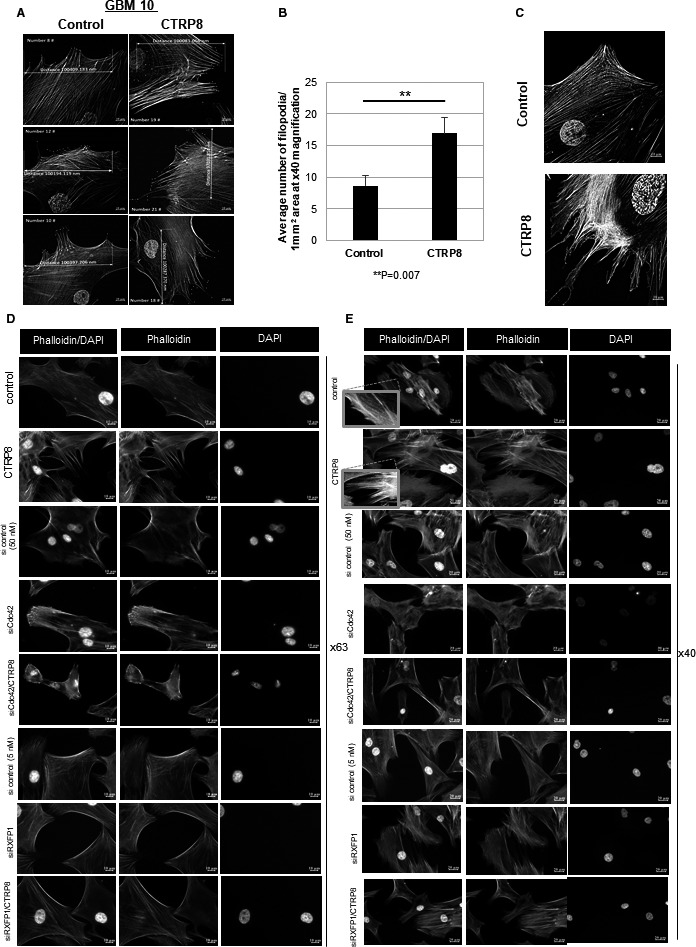
CTRP8 changes the F‐actin cytoskeletal phenotype. Structured illumination (SIM) imaging of filopodia in GBM cells was done with a 40× oil immersion objective (10 µm scale bar). Representative images of three different GBM10 cells are shown, comparing nontreated with CTRP8‐treated (24‐h) GBM cells (A). zen 2.3 lite software (Zeiss) was used to count filopodia in a defined 1‐mm^2^ plasma membrane area. The graph shows average number of filopodia/cell counted in a 1‐mm^2^ plasma membrane area. The results were obtained from three independent experimental data sets (*n* = 3, SEM) that compared 50 randomly selected individual GBM10 cells for CTRP8‐treated (24‐h) and nontreated controls, respectively (B). Representative SIM images (magnification: 40×) demonstrated F‐actin‐containing membrane extensions upon CTRP8 treatment in GBM10 (C). Representative immunofluorescence images (magnifications shown at 63× and 40×) labeled with phalloidin‐AF594 to detect F‐actin filaments, with cell nuclei stained with DAPI (D, E). CTRP8 treatment for 24 h resulted in an altered F‐actin cytoskeletal phenotype with thin filopodial membrane extensions (D, E); see also inserts (E). This cytoskeletal phenotype was dependent on RXFP1 and Cdc42 and abolished by co‐treatment of GBM cells with CTRP8 and siRXFP1 or siCdc42 (D, E).

**Fig. 4:**
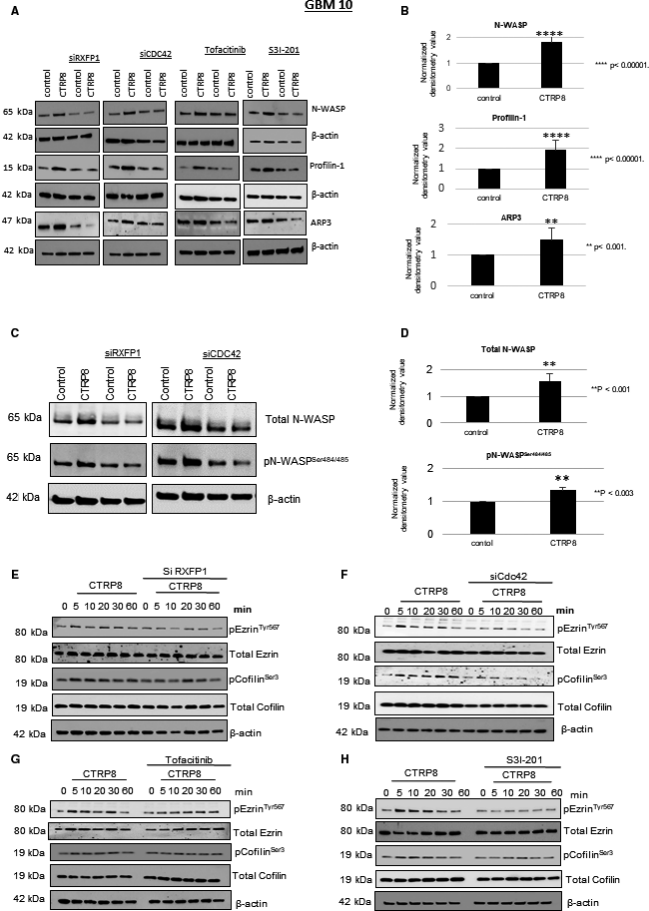
CTRP8 promotes F‐actin nucleation, elongation, and filopodia formation. CTRP8 treatment resulted in the upregulation of N‐WASP, ARP3, and profilin‐1, as shown by western blot analysis (A) and densitometry analysis (B) in patient GBM10 cells. Densitometry graphs represent four independent measurements (*n* = 4) and mean with SD. Combined treatments of CTRP8 with specific siRNAs for RXFP1 and Cdc42 or the inhibitors tofacitinib (10 µm) and S3I‐201 (30 µm) (A) abolished the CTRP8‐mediated increase in Cdc42 cellular protein content of the three actin remodeling factors as shown by western blot (A). Representative western blots of total and active N‐WASP^Ser484/485^ protein in GBM10 cells. Level of total and phosphorylated N‐WASP in GBM cells increased in the presence of CTRP8, and this increase in total N‐WSAP and N‐WASP^Ser484/485^ was diminished in the presence of siRXFP1 and siCdc42 (C). Graphs represent the averages from three independent western blot experiments (D). CTRP8 promoted the phosphorylation of ezrin at Y^567^ and cofilin at S^3^ as shown by western blot detection (E–H). Phosphorylation of ezrin and cofilin was blocked by treatment with siRXFP1 (E), siCdc42 (F), tofacitinib (G), and S3I‐201 (H). Three independent experiments were performed, and beta‐actin was used as a loading control.

Dysregulation of actin filament remodeling impacts cancer cell growth, adhesion, migration, and tissue invasion and attributes poor patient prognosis and treatment failure [64, 65, 66]. Employing fluorescence imaging and super‐resolution SIM, we investigated the effect of CTRP8 on actin cytoskeletal remodeling processes that may facilitate enhanced motility in GBM cells. We used Alexa 594‐labeled phalloidin to visualize F‐actin polymers and quantify filopodia (Fig. 3A,B; Fig. S4A–D). CTRP8 treatment for 24 h resulted in the formation of elongated F‐actin filaments protruding as filopodia from the cell surface of GBM cells, as shown by fluorescence super‐resolution SIM (Fig. 3A,C) and fluorescence bright‐field microscopy (Fig. 3D,E; Fig. S4B,D). F‐actin remodeling in response to CTRP8 resulted in an approx. 50% increase in filopodia formation (Fig. 3B), and this was dependent on the presence of RXFP1 and Cdc42. When exposed to CTRP8, GBM cells treated with siRXFP1 and siCdc42 failed to form filopodial membrane extensions and resembled untreated controls (Fig. 3D,E). To study the new role of the CTRP8‐RXFP1‐Cdc42 axis in F‐actin cytoskeletal remodeling, we investigated molecular mechanisms in the multistep F‐actin filament formation process that may be affected by CTRP8. Recruitment and activation of Cdc42 lead to the activation of N‐WASP and the formation of the N‐WASP‐Arp2/3 nucleation complex, which recruits G‐actin monomers for actin filament elongation, while actin filament branching is controlled by profilin‐1 [67]. CTRP8 treatment for 24 h caused an increase in protein content of N‐WASP by 1.3‐fold, Arp3 by 1.7‐fold, and profilin‐1 by 2.7‐fold in patient GBM and U87MG cells (Fig. 4A,B; Fig. S5A–D). This upregulation of N‐WASP, Arp3, and profilin‐1 was abolished upon silencing of RXFP1 and Cdc42 (Fig. 4A,C; Fig. S5A,C). Similarly, treatment with tofacitinib or S3I‐201 blocked the CTRP8‐mediated upregulation of N‐WASP, Arp3, and profilin‐1 (Fig. 4A; Fig. S5A,C). In addition, we showed that CTRP8 increased the cellular level of active N‐WASP^S484/485^, which is a critical component for actin nucleation and key factor for filopodia formation (Fig. 4C,D) [68]. The F‐actin barbed‐end binding protein Ena/VASP and its interaction partner WAVE aid Arp2/3 complex in mediating actin polymerization, promote filopodia formation, and enhance cell migration [69, 70]. We found that the CTRP8‐RXFP1‐Cdc42 axis did not affect cellular protein levels of WAVE or Ena/VASP and CTRP8 had no effect on the phosphorylation status of VASP at residue Ser157 in GBM10 cells (Fig. S5E,F).

**Fig. 5:**
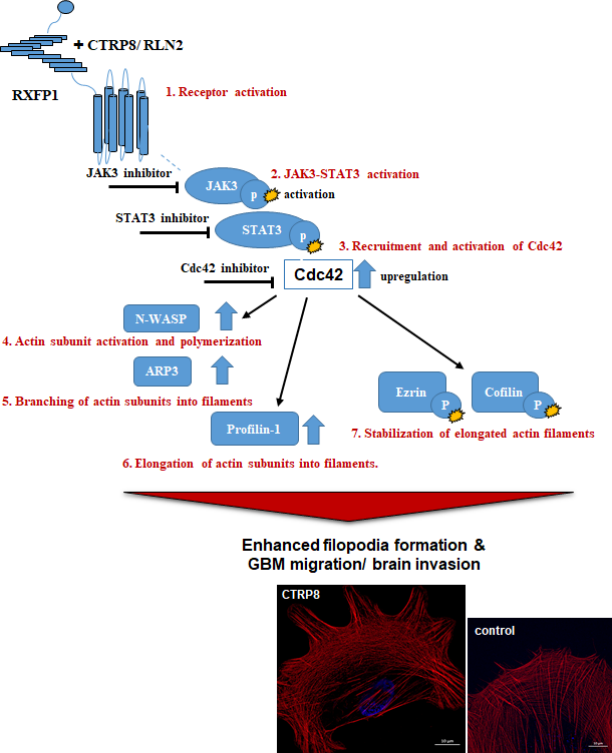
Schematic diagram of the CTRP8‐RXFP1‐JAK3‐STAT3‐Cdc42 signaling axis. Graphical sketch of the activation of RXFP1 by CTRP8 ligands (CTRP8 or RLN2) and downstream activation of the JAK3‐STAT3 axis with enhanced Cdc42 activation and cellular protein content. This resulted in Cdc42‐dependent upregulation of N‐WASP, ARP3, and profilin‐1 to promote nucleation, branching, and elongation of F‐actin filaments. The CTRP8‐RXFP1‐JAK3‐STAT3‐Cdc42 actin remodeling cascade also stabilized elongated actin filaments through phosphorylation of ezrin and cofilin. Functionally, the CTRP8‐RXFP1 affected key steps in the actin cytoskeletal dynamics to translate into enhanced GBM cell motility and filopodia formation. Specific JAK3 and STAT3 inhibitors abolished this cytoskeletal response and may be attractive drug targets in preventing GBM invasion.

We showed that the CTRP8‐RXFP1‐JAK3‐STAT3 signaling cascade alters actin cytoskeletal remodeling dynamics in patient GBM cells by enhancing the protein content and activity of Cdc42 and other key factors involved in actin filament formation. However, filopodia formation requires F‐actin filaments to associate with the inner cell membrane, a process facilitated by the ezrin, radixin, and moesin (ERM) proteins and requiring the phosphorylation of ezrin [71, 72]. Activation of the CTRP8‐RXFP1 cascade coincided with an early phosphorylation of ezrin^Thr567^ in GBM cells (Fig. 4E–H; Fig. S6A–F). CTRP8 treatment also resulted in phosphorylation of the actin filament disassembly factor cofilin at Ser3, a residue causally linked to the inhibition of F‐actin filament severing activity of cofilin. The CTRP8‐mediated phosphorylation of both ezrin and cofilin was diminished by siRXFP1 and siCdc42 treatment in patient GBM and U87MG cells (Fig. 4E,F; Fig. S6A,B,E). Phosphorylation of ezrin and cofilin also required active JAK3‐STAT3 signaling and abolished upon treatment with tofacitinib and S3I‐201 (Fig. 4G,H; Fig. S6C,D,F). The cognate RXFP1 ligand RLN2 replicated the molecular F‐actin remodeling phenotype we had observed with CTRP8. Human recombinant RLN2 increased the protein content of Cdc42 (Fig. S1G), N‐WASP, and profilin‐1 (Fig. S7A), and the increase in protein content of WAVE and profilin‐1 was diminished by siCdc42 treatment in GBM10 (Fig. S7A). Like CTRP8, RLN2 increased F‐actin polymerization with prominent filopodia formation as determined by phalloidin fluorescence imaging (Fig. S7B,C) and enhanced GBM motility (Fig. S3I,J). The results of this study are summarized schematically (Fig. 5) and identified the small Rho GTPase Cdc42 as a new target of a RXFP1‐JAK3‐STAT3 signaling cascade activated by the RXGP1 agonists CTRP8 and RLN2. This resulted in enhanced abundance and activity of Cdc42 and promoted several key steps in F‐actin remodeling, filopodia formation, and migration of GBM (Fig. 5).

## 4 Discussion

The activation of the G protein‐coupled receptor RXFP1 by RLN2 or CTRP8 has been associated with enhanced tumor cell motility and tissue invasion in several tumors, but the underlying molecular mechanisms remain poorly understood [55, 73, 74, 75, 76, 77, 78]. Here, we have identified RXFP1 as a new member of a growing number of GPCRs that utilize the JAK‐STAT signaling pathways previously associated with cytokine and growth factor receptor signaling during development, inflammation, and tumorigenesis [61, 79, 80], including glioma [81]. We identified the intracellular kinase JAK3, a member of the JAK family preferentially expressed in hematopoietic cells [82], as a specific downstream target of activated RXFP1. JAK3 was shown to be critical for STAT3 phosphorylation in patient GBM cell isolates. Heterotrimeric Gα proteins had previously been shown to bind and phosphorylate JAK, thus linking GPCR–ligand interaction with JAK‐STAT pathway activation [79, 83]. A major focus in glioma has been on aberrant JAK2‐STAT3 pathway activation, which enhances GBM tumorsphere formation and invasiveness in adult malignant glioma grade III (anaplastic astrocytoma), grade IV (glioblastoma), and the most common pediatric brain tumor medulloblastoma [84, 85, 86, 87]. Inhibition of JAK2 activity with AG490 [88, 89], WP1066 [90], sorafenib [91], and WP1193 [92] downregulated STAT3 activity, inhibited proliferation, migratory/ invasive behavior, and focal adhesion kinase signaling of glioma cell lines, and resulted in apoptosis [93]. JAK2 inhibitors were also shown to improve the survival of mice orthotopically xenografted with patient GBM brain tumor‐initiating cells and treated with TMZ [94]. However, the ability of RXFP1 to engage the JAK3‐STAT3 signaling pathway provides a hitherto underappreciated new route of promoting GBM invasiveness and, in part, may explain the limited treatment success of JAK2 inhibitors in malignant glioma.

While members of the RhoA family of small GTPases have long been known as key regulators of actin remodeling and focal adhesion assembly [33], our understanding of their functional roles in glioma cell motility and the insidious brain tissue infiltration of GBM cells is still emerging [95]. Elevated expression of Cdc42 has been linked to increased GBM progression with high‐tissue invasiveness, shorter progression‐free survival in mice [40], and poor prognosis of glioma patients [39, 95]. We show that the ability of both RXFP1 agonists, CTRP8 and RLN2, to enhance the motility of patient GBM cell was critically dependent on cellular Cdc42. CTRP8 induced a marked shift from inactive to active GTP‐bound Cdc42 in human glioma cells, indicating a novel role of CTRP8 in regulating Cdc42 activity. Disruption of the RXFP1‐JAK3‐STAT3 signaling cascade by specific KD or pharmacological inhibition abolished the CTRP8‐mediated increase in cellular Cdc42 protein levels and reduced the motility of patient GBM cells. Cdc42 KD did not affect CTRP8‐induced phosphorylation of either JAK3 or STAT3, indicating downstream STAT3 activation of Cdc42 in our GBM models. Human pancreatic cancer cells were shown to utilize IL‐6‐mediated Jak2‐STAT3 signaling to form a trimeric complex of Cdc42 with its interaction partner IQGAP1 and STAT3 for IQGAP‐mediated activation of Cdc42 [96]. IQGAP1 protein was present in our GBM cell models, but contrary to Cdc42, the protein levels of IQGAP1 remained unchanged upon CTRP8 treatment (data not shown). It is likely that a functional JAK3‐STAT3‐IQGAP1‐mediated Cdc42 activation pathway exists in human glioma cells. The consistent upregulation of Cdc42 protein upon CTRP8 treatment is unlikely the result of STAT3 transcriptional activation of the Cdc42 gene, since Cdc42 is not a known STAT3 target gene [97] and CTRP8 did not alter Cdc42 mRNA expression levels in our GBM cells (data not shown). How CTRP8‐RXFP1‐JAK3‐STAT3 signaling mediates the increase in Cdc42 protein remains to be determined and may involve post‐translational modifications [98, 99, 100, 101].

The pro‐migratory activity of the CTRP8‐RXFP1‐JAK3‐STAT3‐Cdc42 axis coincided with a significant increase in filopodia formation in patient GBM cell isolates. Serving as a morphological hallmark of Cdc42 activation, filopodia and microspikes are needle‐like actin‐rich membrane extensions with mechanical and sensory properties that are critical for the directionality of migrating cells [102, 103, 104]. As shown for Cdc42, the activation of the CTRP8‐RXFP1‐JAK3‐STAT3 pathway increased the cellular protein content of N‐Wiskott–Aldrich syndrome (N‐WASP; 1.3×), Arp3 (1.7×), and profilin‐1 (2.7×), which are key factors facilitating *de novo* polymerization of a dynamic actin core for the staged process of filopodia nucleation and elongation [103]. GTP‐Cdc42 interacts with and activates Arp2/3 complex through N‐WASP to link GTPase activation with actin cytoskeletal remodeling [105]. The multifunctional Cdc42‐WASP‐Arp2/3 complex acts as a filopodial actin nucleator, attracts profilin‐1‐bound G‐actin monomers to fuel the elongation step at fast‐growing F‐actin barbed ends, and antagonizes actin barbed‐end capping proteins [106, 107]. The interaction of N‐WASP with Cdc42 is critical for filopodia formation and cells expressing an N‐WASP with a mutated Cdc42 binding site are unable to form filopodia, despite the presence of intact Cdc42 [108]. CTRP8 signaling did not affect the actin filament cross‐linking protein fascin, which binds actin filament pairs to act as precursors for filopodia formation [109]. Intriguingly, we observed increased phosphorylation of cofilin upon RXFP1 activation which indicates an inactivation of cofilin and its actin severing activity. This stabilizes F‐actin fibers and reduces the destabilizing effect of dephosphorylated/ active cofilin on fascin‐cross‐linked F‐actin bundles in filopodia [110]. Enhanced F‐actin assembly and elongation leading to increased filopodia formation and motility of GBM are clinically relevant phenotypes of the Cdc42‐mediated cytoskeletal signature activated by the novel CTRP8/RLN2‐RXFP1‐JAK3‐STAT3 cascade in human glioma.

As a downstream target of prenylated Rac GTPase and acidic phospholipids, the WAVE‐Ena/VASP complex activates the Arp2/3 complex to promote actin nucleation in motile cells [111, 112]. Notably, CTRP8 failed to alter the cellular protein content of WAVE and its interaction partner Ena/VASP. Furthermore, CTRP8 did not enhance phosphorylation of Ena/VASP, thus endorsing the view that CTRP8‐RXFP1‐JAK3‐STAT3 pathway can promote filopodia formation by selectively targeting the Cdc42‐WASP‐Arp2/3 complex and profilin‐1 for enhanced actin nucleation and elongation. The Cdc42‐mediated propulsive dynamic actin remodeling mechanism, combined with our report on the upregulation of proteolytic cathepsin B upon RXFP1 activation [45], establishes the CTRP8‐RXFP1 system as a powerful new promoter of GBM invasiveness. Furthermore, regional GBM tissue‐based RNAseq data (http://glioblastoma.alleninstitute.org/) showed enrichment of both RXFP1 and Cdc42 transcripts at the invading front in patient GBM tissues. Hence, the CTRP8‐RXFP1‐Cdc42 axis emerges as an important new functional player in GBM invasion. Our findings also suggest additional oncogenic roles for the CTRP8‐RXFP1‐JAK3‐STAT3‐Cdc42 pathway. CTRP8 caused a Cdc42‐dependent enhancement in phosphorylation of ezrin, a member of the ERM family, connecting the plasma membrane with the underlying actin cytoskeleton to stabilize filopodia. Cdc42 GTPase activity was shown to elevate levels of activated ezrin at filopodial tips, and this contributes to the cellular transformation of Fos‐transformed fibroblasts [113]. The oncogenic potential of phosphorylated ezrin includes its ability to serve as an important topological organizer of specialized membrane domains to enable potent oncogenic signaling functions in tumor cells [114, 115, 116].

## 5 Conclusions

We provide evidence for an important new role of a novel CTRP8‐RXFP1‐JAK3‐STAT3‐Cdc42 axis in targeting F‐actin assembly and filopodia formation to promote tumor cell invasion in patient GBM.

## Author contributions

TT and AG performed the experimental work. JB and MP assisted in tissue and clinical data collection and provided critical review of the manuscript. SH‐K and TK conceived the study. TK lead the study and drafted the manuscript. All authors approved of the final version of this manuscript prior to submission.

## Conflict of interest

The authors declare no conflict of interest.

## Supporting information


**Fig. S1**. RXFP1 facilitates CTRP8‐mediated phosphorylation of JAK3 and STAT3. Treatment with CTRP8 (100 ng·mL^−1^; 0–60 min) resulted in increased phosphorylation of JAK3^Y0980/981^ and STAT3^Y705^ as determined by Western blot of total cell lysates of U87MG (A, B) and GBM146 (C–F). SiRXFP1 treatment (100 nm) blocked this CTRP8‐mediated activation of the JAK3‐STAT3 pathway as shown for JAK3^Y0980/981^ and STAT3^Y705^ detection in U87MG (A) and STAT3^Y705^ in GBM146 (C). Both, S3I‐201 (30 µm; B, F) and tofactinib (10 µm; E) abolished phosphorylation of STAT3 in U87MG (B) and GBM146 (F). When tested in GBM146, siCdc42 (50 nm) did not affect CTRP8‐mediated increase in STAT3^Y705^ levels indicating that Cdc42 was located downstream of STAT3 (D). Total JAK3 and STAT3 and beta‐actin served as loading controls Treatment of GBM10 with human recombinant RLN2 for 24 h resulted in an up‐regulation of total Cdc42 protein that was blocked by siRXFP1, Tofacitinib, and S3I‐201 as shown in representative Western blots (G). Like CTRP8, RLN2 also caused early and strong activation of STAT3 and the level of STAT3^Y705^ phosphorylation was dependent on RXFP1 in GBM10 (H). Representative Western blots of three independent experiments are shown.

**Fig. S2**. No effect on protein levels by DMSO solvent control and control siRNA. Upon treatment of GBM10 with control siRNA (A‐C) and DMSO used at a concentration to dissolve STAT3 and JAK3 inhibitors (A, D, E), representative Western blots demonstrated that treatments with control siRNA or DMSO had no effect on the protein levels of Cdc42 (A) and total/ phospho‐protein levels of STAT3/ STAT^Tyr705^, cofilin/ cofilin^Ser3^, and ezrin/ ezrin^Tyr567^ (B–E). Representative examples of gene silencing of RXFP1 and Cdc42 in the three different patient GBM cell lines employed in this study (F, G).

**Fig. S3**. CTRP8 enhanced motility of GBM cells is RXFP1 and Cdc42‐dependent. CTRP8 treatment enhanced the motility of RXFP1^+^ patient GBM34 (A–D) and GBM146 (E–H) over a 10 h observation period, albeit at different trajectories, as determined by real‐time migration assays. Treatment with siRXFP1 (5 nm; A, B, E, F) or siCdc42 (50 nm; C, D, G, H) resulted in a decrease in GBM motility, as shown for GBM34 (A–D) and GBM146 (E–H). Each experiment was performed as two independent duplicates for each treatment. RLN2 significantly enhanced GBM motility as determined in real‐time migration assays (I, J). Data are represented as a mean value with a *P*‐value of < 0.001 (***) and < 0.00001 (****); NS=Not Significant. WST assay showed no significant changes in metabolic activity in GBM cells upon treatment with JAK3i, STAT3i, and DMSO solvent control compared to non‐treaded control cells (K).

**Fig. S4**. CTRP8 reorganizes the F‐actin cytoskeleton in patient GBM cells. We investigated the effect of CTRP8 treatment (24 h) on the F‐actin cytoskeleton using phalloidin‐ Alexa Fluor‐594 labeling and immunofluorescence imaging of GBM34 (A, B) and GBM146 (C, D). Images were taken using x40 magnification with a Zeiss Z2 microscope system. Both GBM cell models responded to CTRP8 with changes in F‐actin cytoskeletal phenotype (B, D). GBM146 responded to CTRP8 with a dramatic increase in filopodia formation when compared to untreated controls (D). DAPI was used as a nuclear stain and representative images are shown.

**Fig. S5**. CTRP8‐RXFP1‐JAK3‐STAT3 pathway increases the cellular protein content of key F‐actin remodeling factors in GBM. Total protein lysates of GBM34 (A, B) and U87MG (C, D) treated with CTRP8 for 24 h showed significantly increased levels of N‐WASP, profilin‐1, and ARP3 when compared to untreated controls. This CTRP8 response was abolished when GBM 34 (A) and U87MG (C) were treated for 24 h with siRXFP1 (100 nm), siCdc42 (50 nm), S3I‐201 (30 µm), and Tofacitinib (10 µm). The CTRP8‐RXFP1‐JAK3‐STAT3‐Cdc42 axis targeted the actin nucleation and elongation complex of N‐WASP/ARP2/3 and profilin‐1. Densitometry graphs of N‐WASP, ARP3, and profilin‐1 plus/ minus CTRP8 treatment for 24 h are shown and represent data collected from three independent experiments. Total protein levels of ARP3, N‐WASP, and profilin‐1 were normalized to beta‐actin for GBM34 (B) and U87MG (D). The *P*‐values of < 0.001(**) and < 0.0001(***) were considered significant. CTRP8 failed to alter the cellular protein content of WAVE, its interaction partner Ena/VASP, and fascin (E) and did not alter phosphorylation of Ena/VASP (F) as shown for GBM10 (E, F). Hence, the actin polymerization promoting WAVE‐Ena/VASP complex was not targeted by an activated CTRP8‐RXFP1 axis to increased GBM motility.

**Fig. S6**. CTRP8‐RXFP1‐JAK3‐STAT3‐Cdc42 axis promotes filopodia formation. Cellular levels of activated membrane‐actin cross‐linker ezrin^Y567^, a key factor promoting the formation of filopodia, were increased upon treatment with CTRP8 in GBM146 (A‐D) and U87MG (E, F). Phosphorylation of ezrin by CTRP8 was critically dependent on RXFP1 (A, E), Cdc42 (B), and blocked by inhibitors to JAK3 (Tofacitinib; C) and STAT3 (S3I‐201; D, F). Thus, we identified the CTRP8‐RXFP1‐JAK3‐STAT3‐Cdc42 axis as a promoter of filopodia formation in GBM. The members of the CTRP8‐RXFP1‐JAK3‐STAT3‐Cdc42 axis were also instrumental in enhancing cellular cofilin^S3^ levels as determined in GBM146 (A–D). Phosphorylation of cofilin at serin residue 3 causes the inactivation of this actin severing factor and stabilizes F‐actin fibers by reducing the dismantling of fascin‐cross‐linked F‐actin bundles in filopodia [103]. Total ezrin and cofilin remained unchanged by the treatments in both GBM146 (A–D) and U87MG (E, F). Beta‐actin served as loading control.

**Fig. S7**. RLN2 utilizes similar signaling pathways and targets the same actin remodeling factors as CTRP8 to increase GBM migration. Specific KD of RXFP1 or Cdc42 diminished the increase in cellular protein content of N‐WASP and profilin‐1 detected upon RLN2 treatment (A). Like CTRP8, RLN2 promoted F‐actin filament formation and increased filopodial extension in patient GBM cells (B). Data are represented as a mean values with a *P*‐value of < 0.00001 (****); NS, Not Significant.

## Data Availability

The data that support the findings of this study are available from the corresponding author [thomas.klonisch@umanitoba.ca] upon reasonable request.
