# Supplementary figures and images for: Novel CTRP8‐RXFP1‐JAK3‐STAT3 axis promotes Cdc42‐dependent actin remodeling for enhanced filopodia formation and motility in human glioblastoma cells
Source: Mol Oncol. 2021 Jun 18;16(2):368–87. doi: 10.1002/1878-0261.12981 (PMC8763656; doi:10.1002/1878-0261.12981)

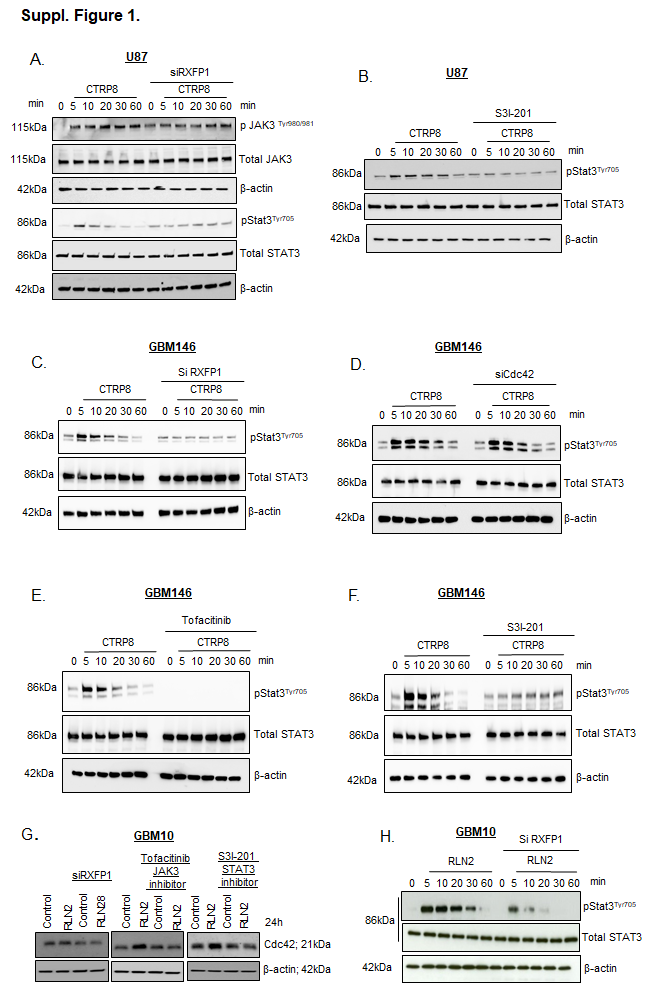

Supplement: Supplementary file 1 — Fig. S1. RXFP1 facilitates CTRP8‐mediated phosphorylation of JAK3 and STAT3. Treatment with CTRP8 (100 ng·mL−1; 0–60 min) resulted in increased phosphorylation of JAK3Y0980/981 and STAT3Y705 as determined by Western blot of total cell lysates of U87MG (A, B) and GBM146 (C–F). SiRXFP1 treatment (100 nm) blocked this CTRP8‐mediated activation of the JAK3‐STAT3 pathway as shown for JAK3Y0980/981 and STAT3Y705 detection in U87MG (A) and STAT3Y705 in GBM146 (C). Both, S3I‐201 (30 µm; B, F) and tofactinib (10 µm; E) abolished phosphorylation of STAT3 in U87MG (B) and GBM146 (F). When tested in GBM146, siCdc42 (50 nm) did not affect CTRP8‐mediated increase in STAT3Y705 levels indicating that Cdc42 was located downstream of STAT3 (D). Total JAK3 and STAT3 and beta‐actin served as loading controls Treatment of GBM10 with human recombinant RLN2 for 24 h resulted in an up‐regulation of total Cdc42 protein that was blocked by siRXFP1, Tofacitinib, and S3I‐201 as shown in representative Western blots (G). Like CTRP8, RLN2 also caused early and strong activation of STAT3 and the level of STAT3Y705 phosphorylation was dependent on RXFP1 in GBM10 (H). Representative Western blots of three independent experiments are shown. [file MOL2-16-368-s007.tif]

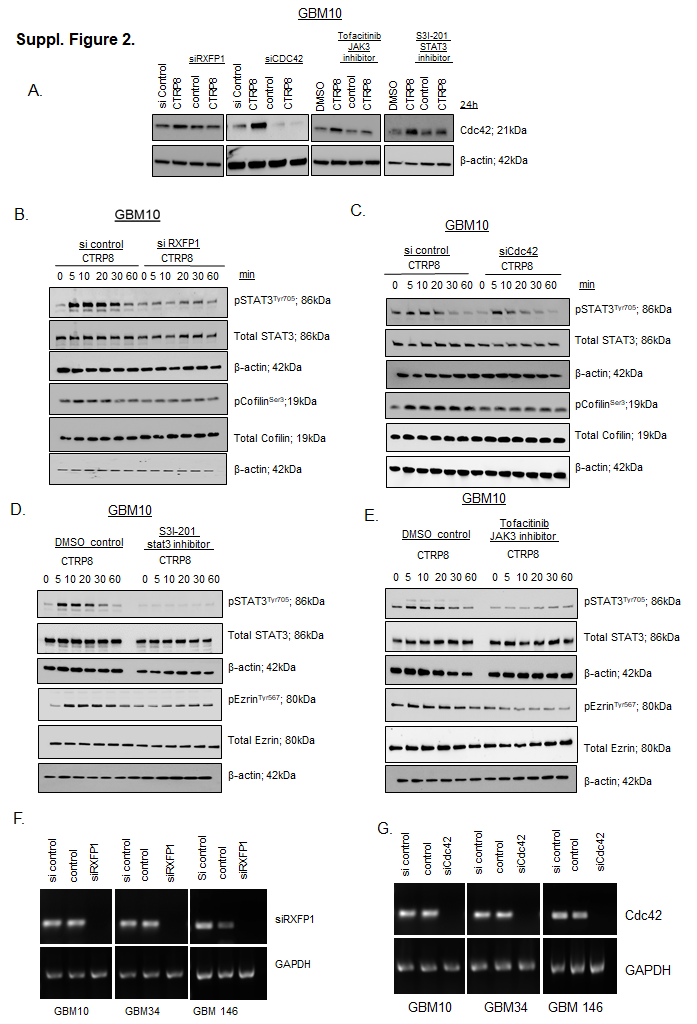

Supplement: Supplementary file 2 — Fig. S2. No effect on protein levels by DMSO solvent control and control siRNA. Upon treatment of GBM10 with control siRNA (A‐C) and DMSO used at a concentration to dissolve STAT3 and JAK3 inhibitors (A, D, E), representative Western blots demonstrated that treatments with control siRNA or DMSO had no effect on the protein levels of Cdc42 (A) and total/ phospho‐protein levels of STAT3/ STATTyr705, cofilin/ cofilinSer3, and ezrin/ ezrinTyr567 (B–E). Representative examples of gene silencing of RXFP1 and Cdc42 in the three different patient GBM cell lines employed in this study (F, G). [file MOL2-16-368-s002.tif]

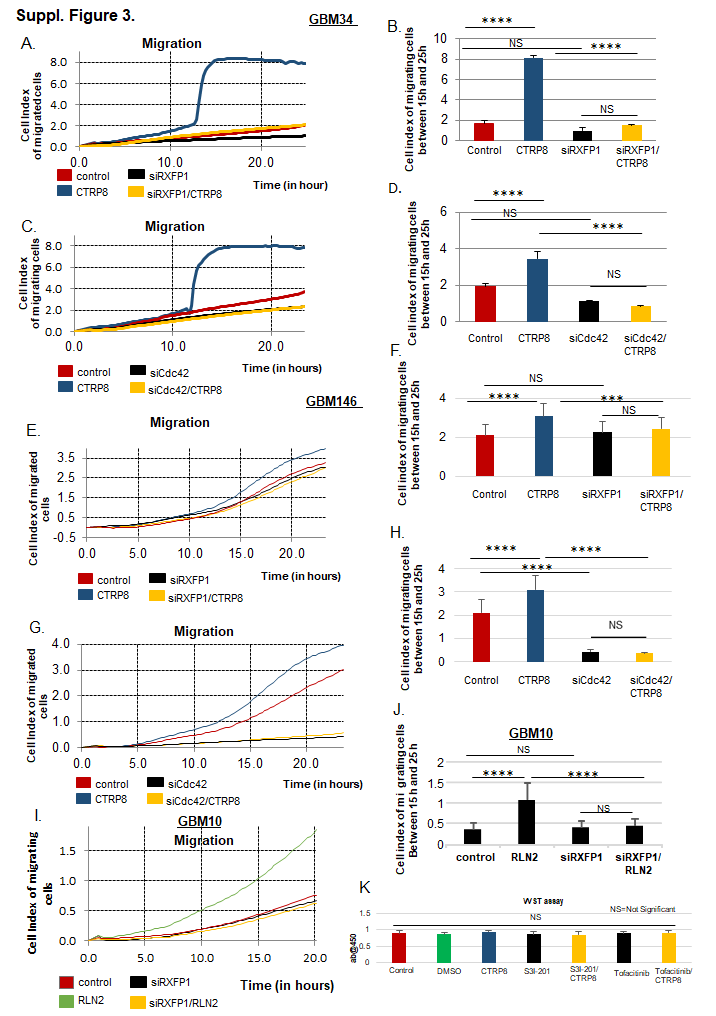

Supplement: Supplementary file 3 — Fig. S3. CTRP8 enhanced motility of GBM cells is RXFP1 and Cdc42‐dependent. CTRP8 treatment enhanced the motility of RXFP1+ patient GBM34 (A–D) and GBM146 (E–H) over a 10 h observation period, albeit at different trajectories, as determined by real‐time migration assays. Treatment with siRXFP1 (5 nm; A, B, E, F) or siCdc42 (50 nm; C, D, G, H) resulted in a decrease in GBM motility, as shown for GBM34 (A–D) and GBM146 (E–H). Each experiment was performed as two independent duplicates for each treatment. RLN2 significantly enhanced GBM motility as determined in real‐time migration assays (I, J). Data are represented as a mean value with a P‐value of < 0.001 (***) and < 0.00001 (****); NS=Not Significant. WST assay showed no significant changes in metabolic activity in GBM cells upon treatment with JAK3i, STAT3i, and DMSO solvent control compared to non‐treaded control cells (K). [file MOL2-16-368-s001.tif]

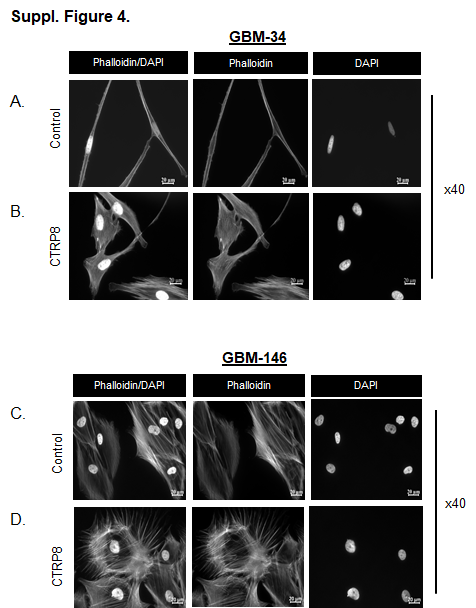

Supplement: Supplementary file 4 — Fig. S4. CTRP8 reorganizes the F‐actin cytoskeleton in patient GBM cells. We investigated the effect of CTRP8 treatment (24 h) on the F‐actin cytoskeleton using phalloidin‐ Alexa Fluor‐594 labeling and immunofluorescence imaging of GBM34 (A, B) and GBM146 (C, D). Images were taken using x40 magnification with a Zeiss Z2 microscope system. Both GBM cell models responded to CTRP8 with changes in F‐actin cytoskeletal phenotype (B, D). GBM146 responded to CTRP8 with a dramatic increase in filopodia formation when compared to untreated controls (D). DAPI was used as a nuclear stain and representative images are shown. [file MOL2-16-368-s005.tif]

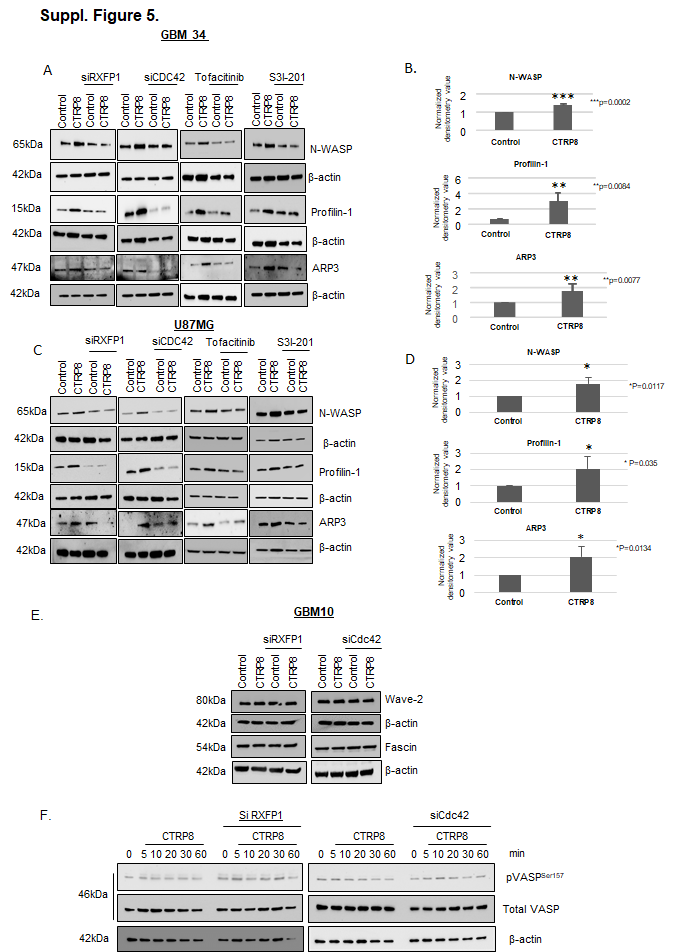

Supplement: Supplementary file 5 — Fig. S5. CTRP8‐RXFP1‐JAK3‐STAT3 pathway increases the cellular protein content of key F‐actin remodeling factors in GBM. Total protein lysates of GBM34 (A, B) and U87MG (C, D) treated with CTRP8 for 24 h showed significantly increased levels of N‐WASP, profilin‐1, and ARP3 when compared to untreated controls. This CTRP8 response was abolished when GBM 34 (A) and U87MG (C) were treated for 24 h with siRXFP1 (100 nm), siCdc42 (50 nm), S3I‐201 (30 µm), and Tofacitinib (10 µm). The CTRP8‐RXFP1‐JAK3‐STAT3‐Cdc42 axis targeted the actin nucleation and elongation complex of N‐WASP/ARP2/3 and profilin‐1. Densitometry graphs of N‐WASP, ARP3, and profilin‐1 plus/ minus CTRP8 treatment for 24 h are shown and represent data collected from three independent experiments. Total protein levels of ARP3, N‐WASP, and profilin‐1 were normalized to beta‐actin for GBM34 (B) and U87MG (D). The P‐values of < 0.001(**) and < 0.0001(***) were considered significant. CTRP8 failed to alter the cellular protein content of WAVE, its interaction partner Ena/VASP, and fascin (E) and did not alter phosphorylation of Ena/VASP (F) as shown for GBM10 (E, F). Hence, the actin polymerization promoting WAVE‐Ena/VASP complex was not targeted by an activated CTRP8‐RXFP1 axis to increased GBM motility. [file MOL2-16-368-s006.tif]

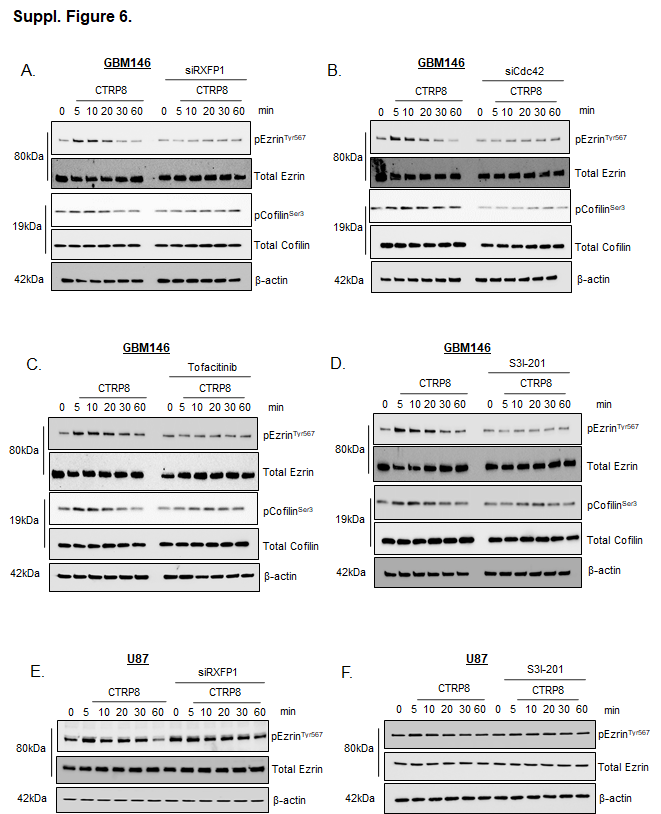

Supplement: Supplementary file 6 — Fig. S6. CTRP8‐RXFP1‐JAK3‐STAT3‐Cdc42 axis promotes filopodia formation. Cellular levels of activated membrane‐actin cross‐linker ezrinY567, a key factor promoting the formation of filopodia, were increased upon treatment with CTRP8 in GBM146 (A‐D) and U87MG (E, F). Phosphorylation of ezrin by CTRP8 was critically dependent on RXFP1 (A, E), Cdc42 (B), and blocked by inhibitors to JAK3 (Tofacitinib; C) and STAT3 (S3I‐201; D, F). Thus, we identified the CTRP8‐RXFP1‐JAK3‐STAT3‐Cdc42 axis as a promoter of filopodia formation in GBM. The members of the CTRP8‐RXFP1‐JAK3‐STAT3‐Cdc42 axis were also instrumental in enhancing cellular cofilinS3 levels as determined in GBM146 (A–D). Phosphorylation of cofilin at serin residue 3 causes the inactivation of this actin severing factor and stabilizes F‐actin fibers by reducing the dismantling of fascin‐cross‐linked F‐actin bundles in filopodia [103]. Total ezrin and cofilin remained unchanged by the treatments in both GBM146 (A–D) and U87MG (E, F). Beta‐actin served as loading control. [file MOL2-16-368-s004.tif]

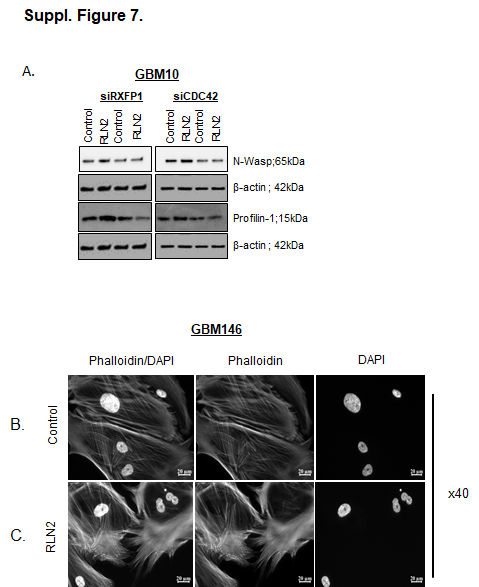

Supplement: Supplementary file 7 — Fig. S7. RLN2 utilizes similar signaling pathways and targets the same actin remodeling factors as CTRP8 to increase GBM migration. Specific KD of RXFP1 or Cdc42 diminished the increase in cellular protein content of N‐WASP and profilin‐1 detected upon RLN2 treatment (A). Like CTRP8, RLN2 promoted F‐actin filament formation and increased filopodial extension in patient GBM cells (B). Data are represented as a mean values with a P‐value of < 0.00001 (****); NS, Not Significant. [file MOL2-16-368-s003.tif]
